# Supplementary material for: Localized translation and sarcomere maintenance requires ribosomal protein SA in mice
Source: J Clin Invest. 2024 May 14;134(13):e174527. doi: 10.1172/JCI174527 (PMC11213506; doi:10.1172/JCI174527)
Supplement: Supplemental data [file jci-134-174527-s032.pdf]

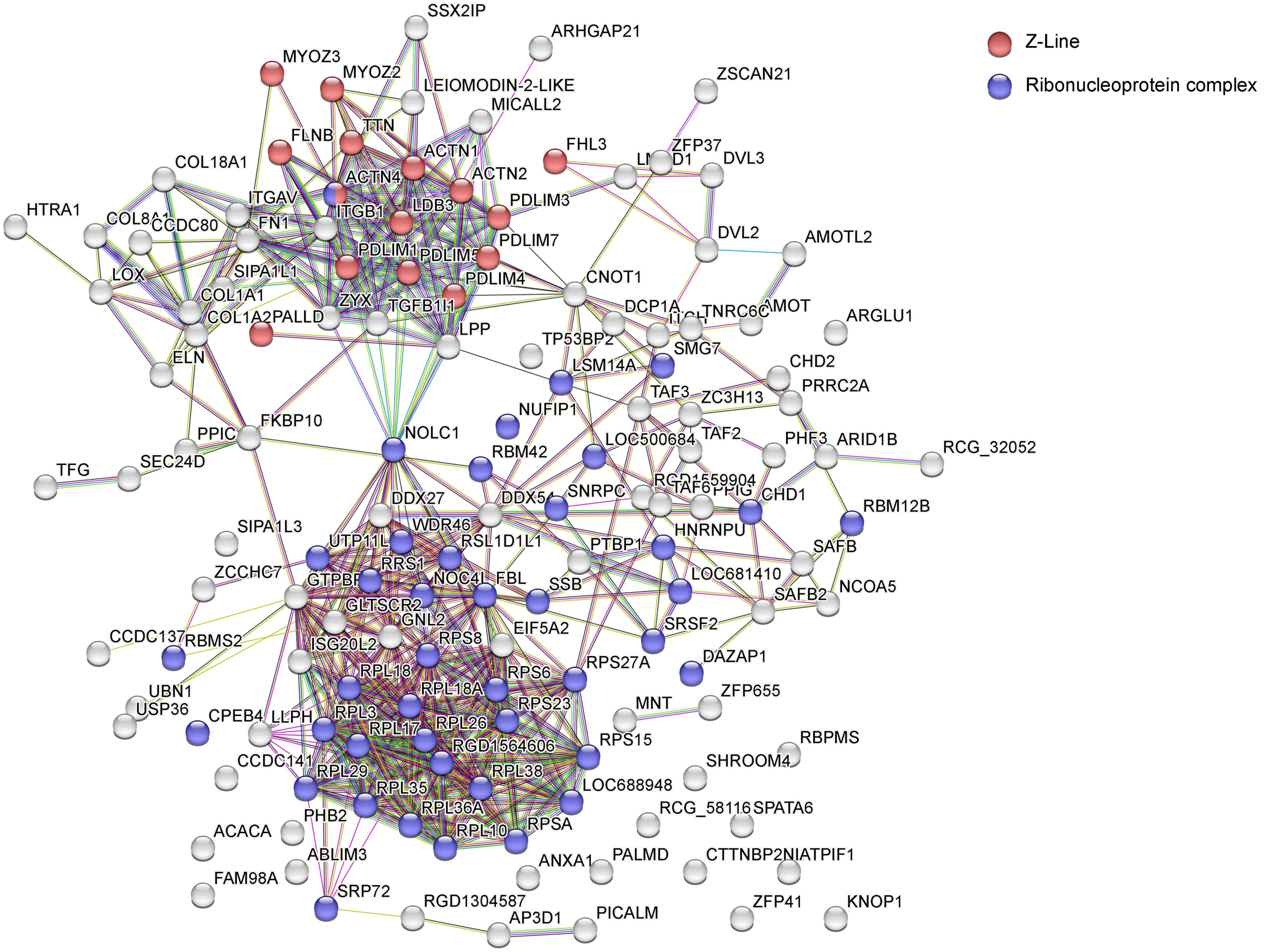

Supplemental Figure 1. STRING of Cypher-BioID2 identified proteins shows Z-line proteins and components of the translational machinery. STRING protein-protein interaction map of proteins significantly enriched by Cypher-BioID2. Proteins labeled in red belong to the Z-line GO term (GO:0030018), FDR for enrichment = 2.61e-11. Proteins labeled in purple belong to the Ribonucleoprotein complex GO term (GO:1990904), FDR for enrichment = 3.83e-16.

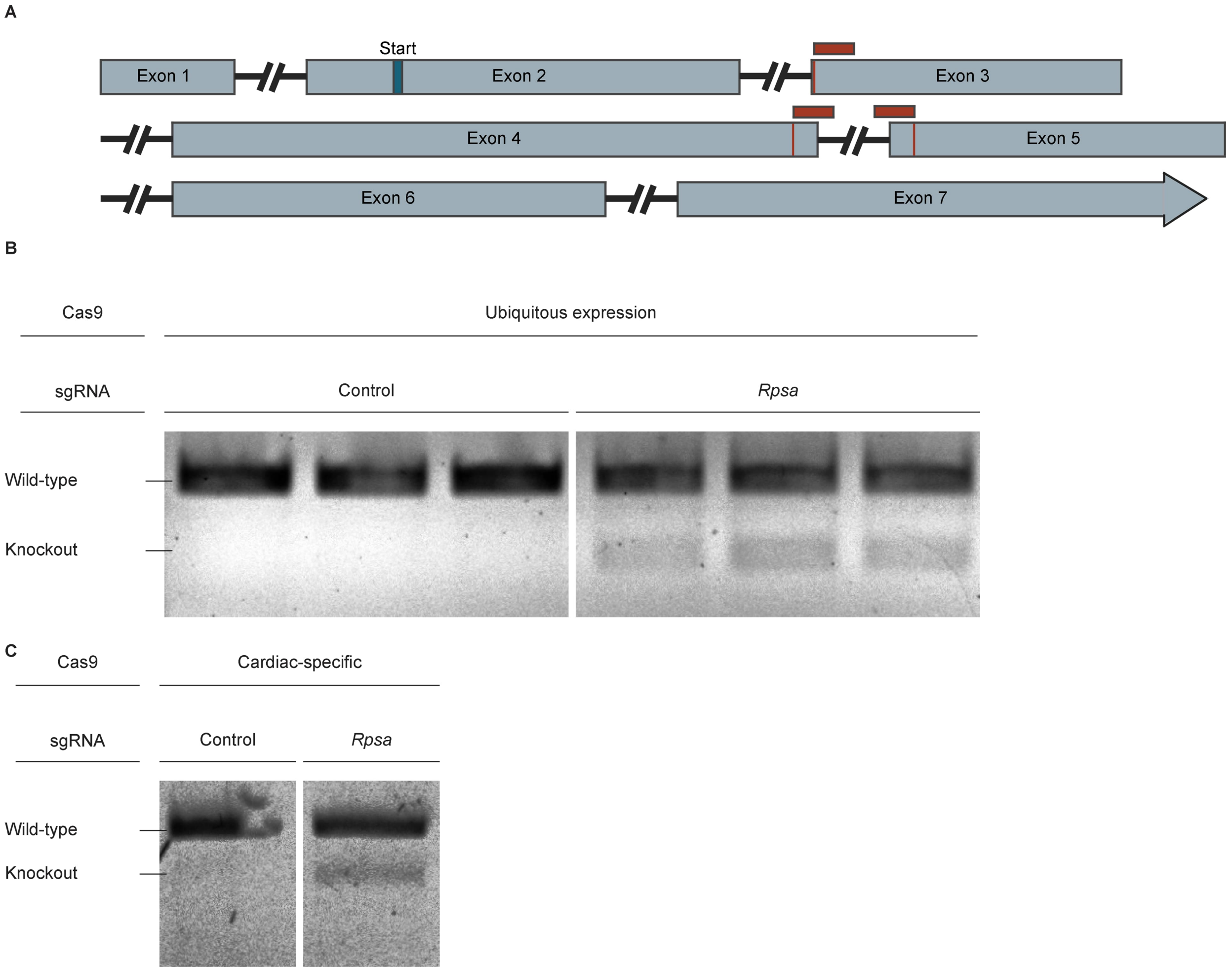

Supplemental Figure 2. Validation of CRISPR-CAS9 knockout of *Rpsa* in vivo. (**A**) Illustration of the *Rpsa* gene with location of sgRNAs targeting exons 3, 4 and 5 (red) and predicted cut sites. (**B** and **C**) T7E 1 assay in mouse heart genomic DNA PCR amplicon surrounding the expected CAS9 cut site in *Rpsa* gene showing successful editing with indels in the *Rpsa* gene in knockout mice. In these mosaic mice we find both edited and unedited cells. The upper band indicates the presence of the wild-type *Rpsa* and the bottom band indicates *Rpsa* with indels. This lower band is observed only in knockout mice transduced with *Rpsa* sgRNA but not with control sgRNA. (**B**) Genomic DNA from control and knockout mice with un-conditional CAS9 expression, and (**C**) with DNA from Lox-stop-Lox-Cas9 mice transduced with troponin driven CRE AAV

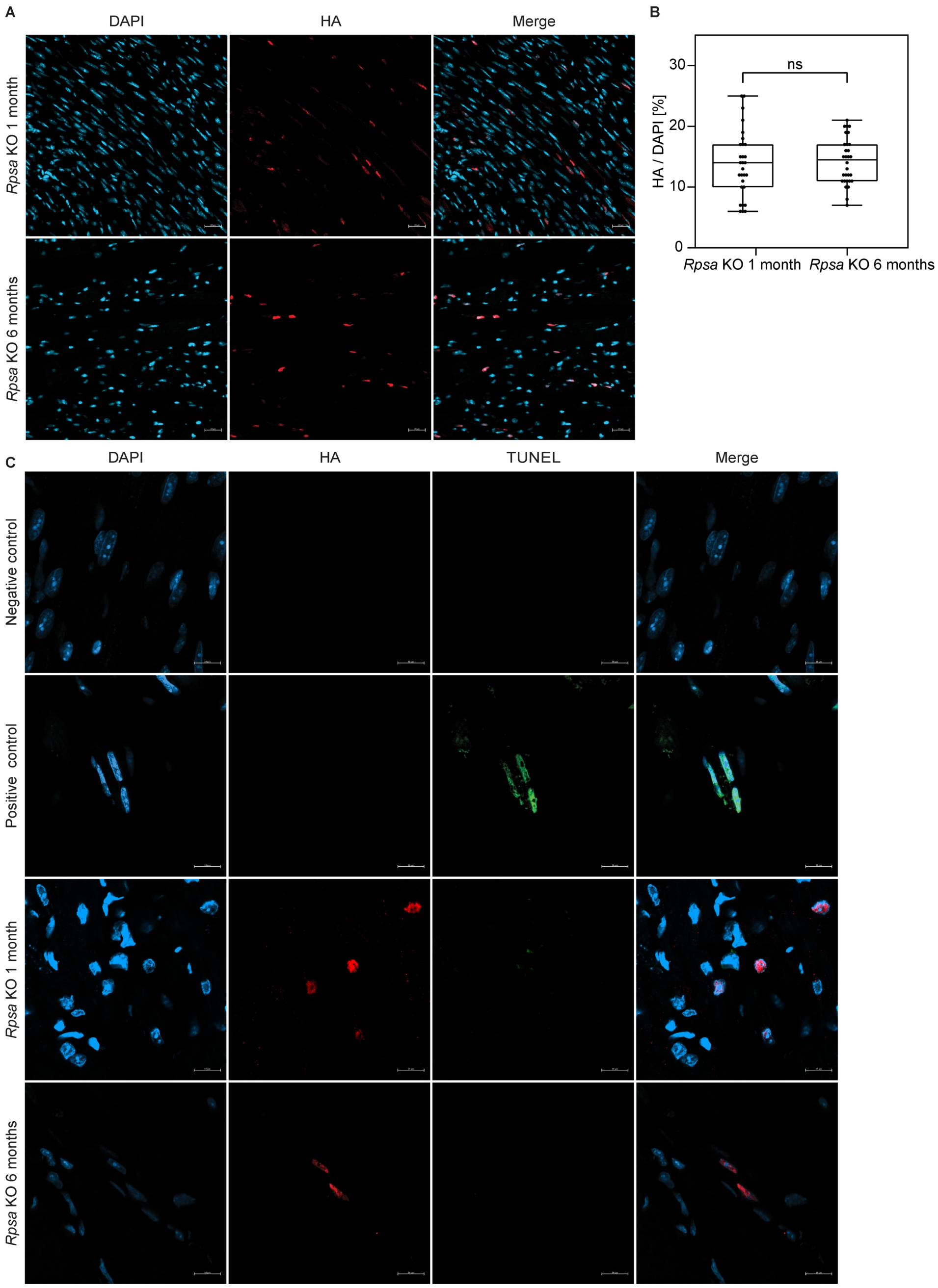

Supplemental Figure 3. Analysis of targeted cell numbers and apoptosis in *Rpsa* knockout hearts. (**A** and **B**) Representative images and quantitative analysis of targeted HA-CRE (red) percentage in *Rpsa* knockout hearts, at 1 and 6 months of age showing no statistically significant difference, nuclei were counter stained with DAPI (blue). Scale bar = 20μm. N = 2-3 mice per experimental group, n = 29, and 30 analyzed images per group. ns ($p > 0.05$), by Student's t test. Data are presented as individual values with box plot displaying the median with 25th and 75th per-centiles. (**C**) Representative images of TUNEL staining of apoptosis. No positive TUNEL (Green) nuclei were detected in *Rpsa* knockout mice, at either 1 or 6 months of age. Positive control is DNase I treated section. Scale bar = 10μm. N = 2-3 mice per group.

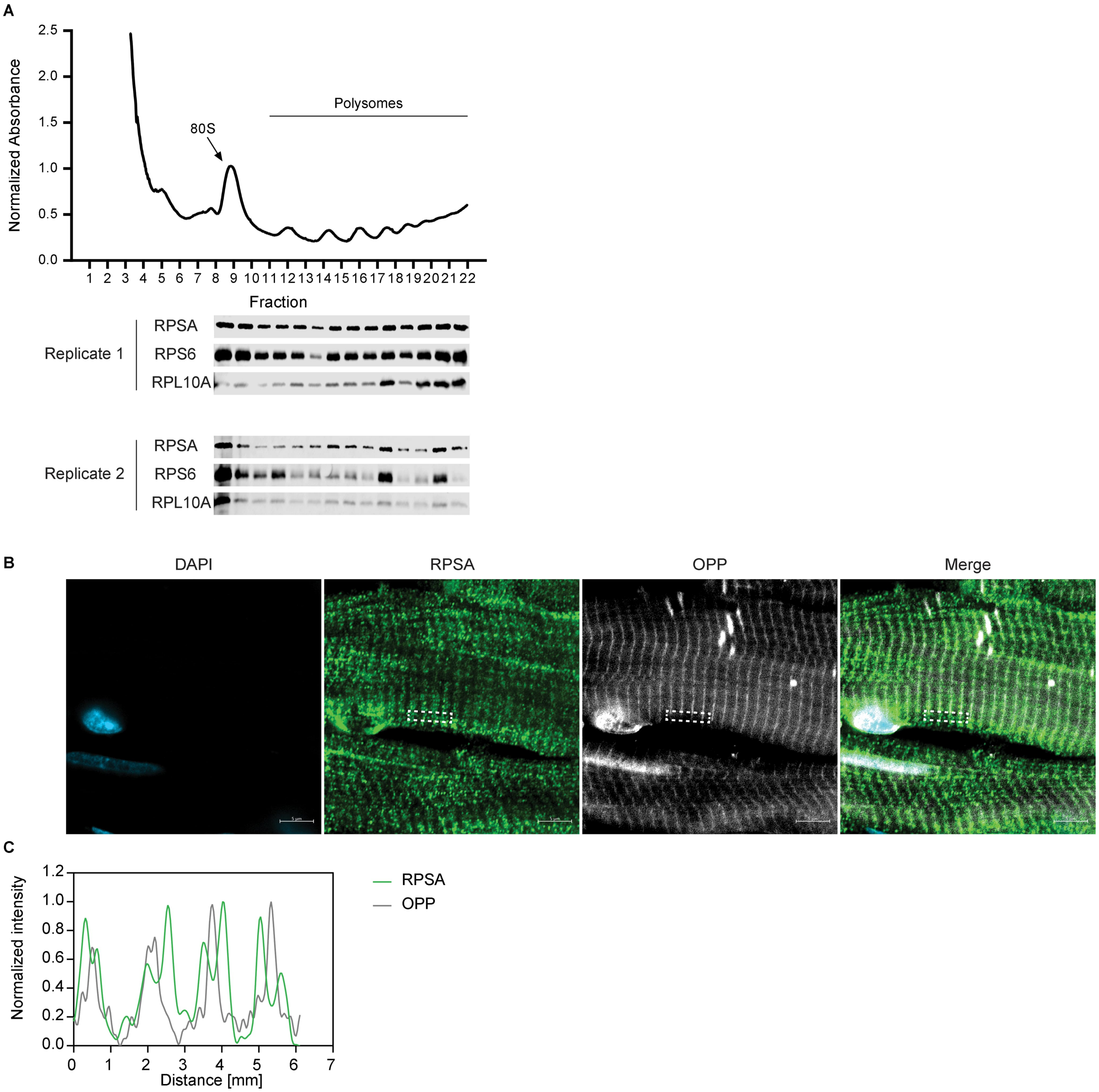

Supplemental Figure 4. RPSA is associated with translating ribosomes. (**A**) Polysome profile and Western blot analyses of normal mouse heart. Western blot of monosome (80S) and polysome fractions were probed with anti-RPSA, anti-RPS6, and anti-RPL10A antibodies showing association of RPSA with translating ribosomes. The experiment was performed in two biological replicates. (**B**) Immunofluorescence images of cardiac sections from mice injected with OPP and stained for OPP (white) and RPSA (green). OPP labeled nascent proteins have a sarcomeric Z-line striated pattern, and RPSA resides in close proximity on both sides of the OPP signal. Scale bar = 5 µm. (**C**) Line scan analysis depicting the prominent, close proximity of RPSA at both sides of OPP labeled nascent proteins.

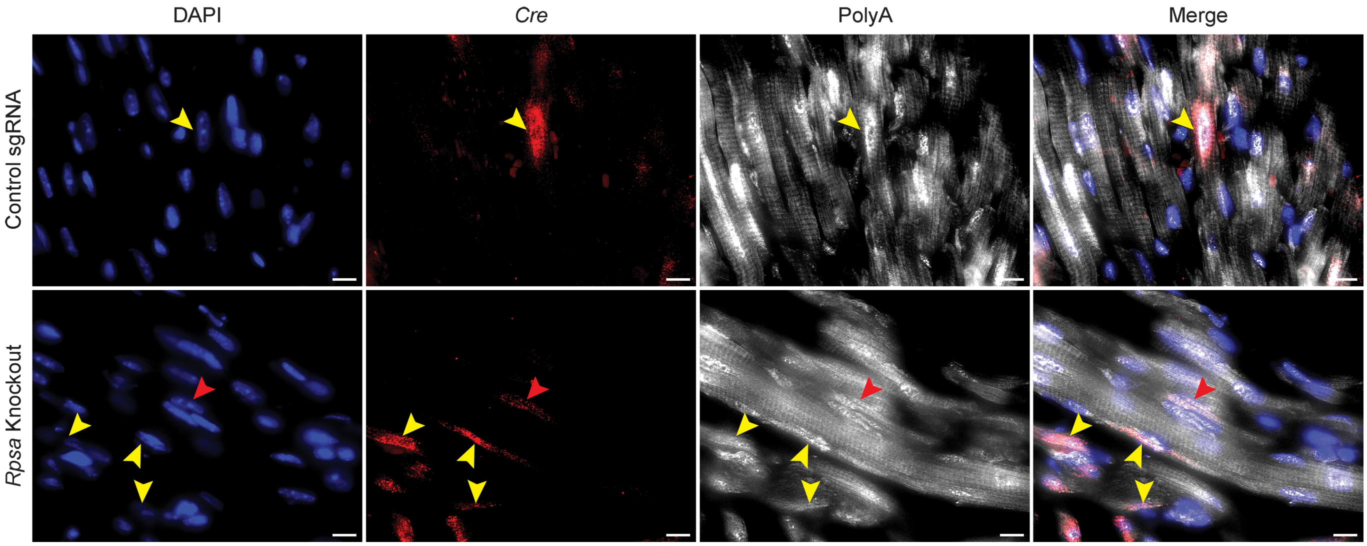

Supplemental Figure 5. *Rpsa* Knockout effect on mRNA distribution. Representative dual smFISH images from *Rpsa* knockout and control hearts incubated with *Cre* (red) and *polyA* (white) probes. In untargeted cells and control sgRNA hearts, the *polyA* mRNA signal exhibits a cross-striated pattern. *Rpsa* targeted cardiomyocytes appear smaller yet show cytoplasmic *polyA* signal. Despite their reduced size, *polyA* striations can be observed in some cells (red arrowhead). Yellow arrowheads indicate *Cre*-positive cells.

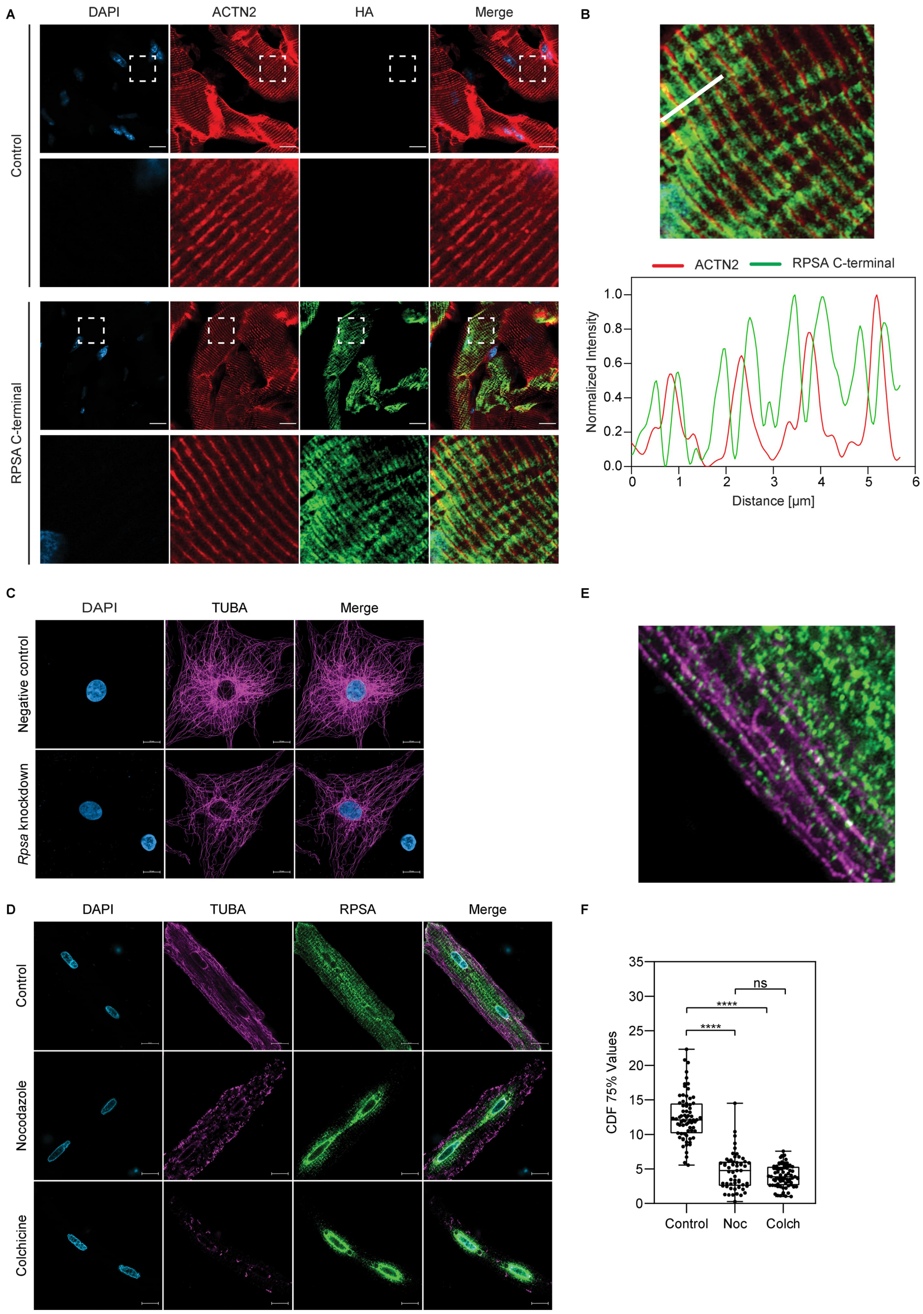

Supplemental Figure 6. RPSA Z-line localization depends on C-terminal fragment and microtubules. (**A** and **B**) Representative images and line scan analysis of cardiac sections from mice transduced with AAV encoding for HA-tagged mCherry-RPSA C-terminal domain. Staining with antibodies for HA (pseudo-colored green) and α-Actinin (pseudo-colored red) shows that the C-terminal domain of RPSA is sufficient for localization to both sides of the Z-line, where endogenous RPSA and where ribosomes are localized. The inset images in (A) are magnified 5 times the original image. The image in (B) is the same inset merged image in (A) and shows the line used for profile analysis. (**C**) Immunofluorescence images for NRVMs stained for Tubulin (TUBA), purple, following *Rpsa* siRNA mediated knockdown or control siRNA (Negative control). While *Rpsa* knockdown did not alter the reticular microtubule pattern, a reduction in total TUBA signal was observed. N = 2 biological replicates. (**D**) Representative images of adult rat cardiomyocytes following microtubule ablation by Nocodazole (Noc) or Colchicine (Colch) showing loss of RPSA's cross-striated localization pattern and a shift to a peri-nuclear localization. (**E**) Higher magnification (8 times the original image) of control cell from D showing intersection of microtubules and RPSA. (**F**) For quantification of RPSA distribution we used the CDF75% measuring the distance in µm from the nucleus in which 75% of the RPSA signal was found, showing peri-nuclear collapse with microtubule ablation. N = 3 biological replicates. n = 55, 70, and 71 analyzed cell per group. ns ($p > 0.05$), **** ($p \leq 0.0001$), by 1-way ANOVA test. Scale bar = 10µm. Data are presented as individual values with box plot displaying the median with 25th and 75th percentiles.

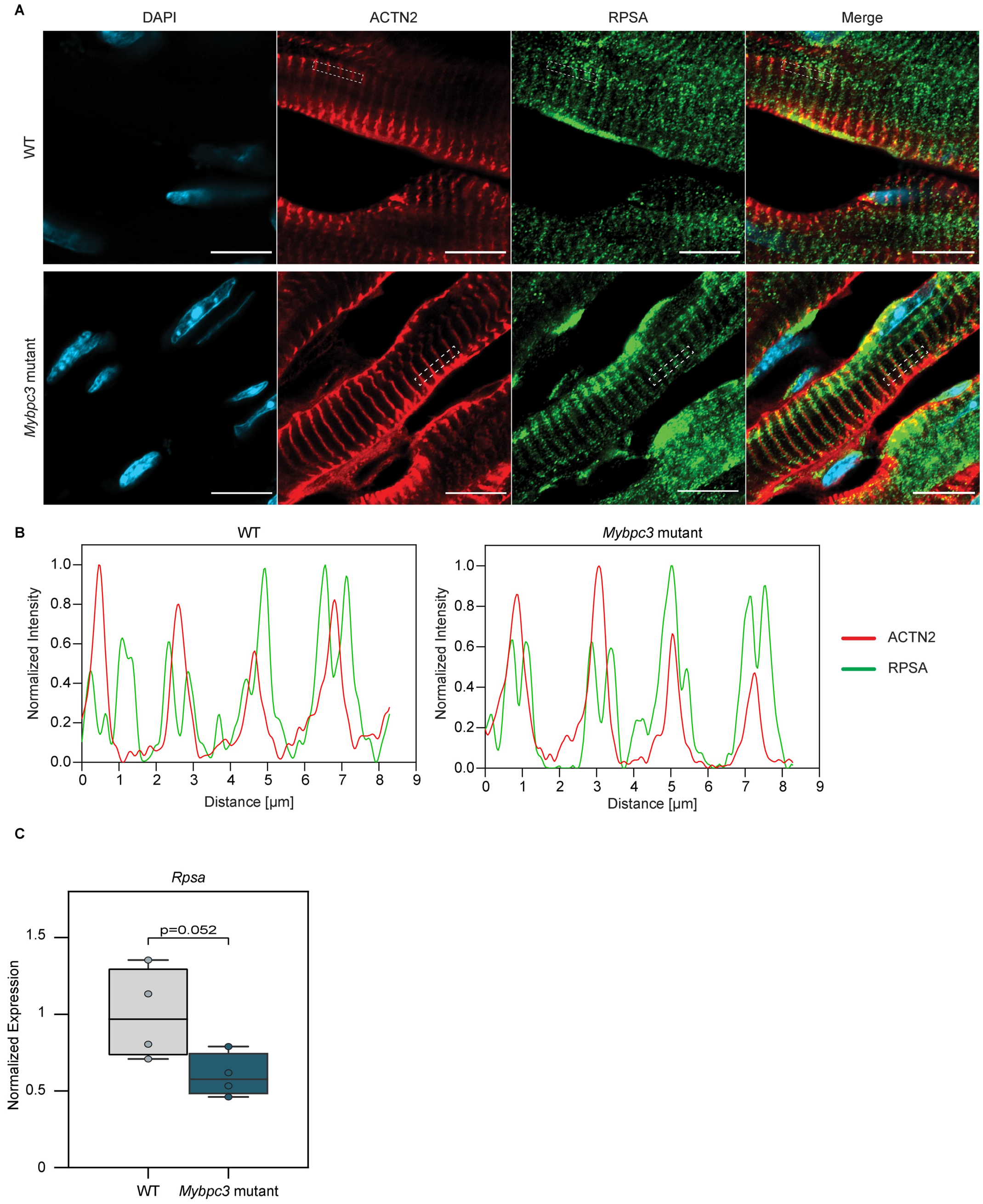

Supplemental Figure 7. The subcellular localization of RPSA and its mRNA expression in *Mybpc3* mutant mice. (**A** and **B**) Representative immunofluorescence images and line scan analysis in Wild Type (WT) and *Mybpc3* mutant mice show unaltered RPSA localization pattern on both sides of the Z disc. RPSA (green), ACTN2 (red), nuclei counterstained with DAPI (blue). N = 3 mice per group. Scale bar = 10µm. (**C**) RT-qPCR analysis of *Rpsa* gene expression in WT and *Mybpc3* mutant mice shows a trend towards reduced *Rpsa* expression (Student's t test , p = 0.052). Expression data was normalized to *Gapdh*. N = 4 mice per group. Data are presented as individual values with box plot displaying the median with 25th and 75th percentiles.

Supplemental Table 1. Identified proteins by Cypher-BioID2 and BioID2 triplicates. The LFQ values for each sample are shown with the Log2 of the fold change and the t-test p value.

Protein Name	Cypher1-BioID2_1	Cypher1-BioID2_2	Cypher1-BioID2_3	BioID2_1	BioID2_2	BioID2_3	log2FC	ttest
LDB3	30.96	30.58	30.69	19.51	19.22	17.80	11.90	0.000
PDLIM4	26.53	26.15	25.94	20.65	19.10	18.28	6.86	0.001
ACTN1	24.68	25.55	25.09	19.36	19.19	16.90	6.62	0.001
ABLIM3	25.19	25.83	24.23	19.13	21.06	16.90	6.05	0.009
VIRMA	25.15	24.99	24.64	19.81	19.57	18.64	5.59	0.000
ACTN4	31.02	31.58	31.23	25.54	26.67	25.13	5.49	0.000
RPL18	26.09	27.09	26.01	21.02	23.27	18.59	5.44	0.018
PDLIM7	29.45	29.80	28.68	24.85	25.36	22.28	5.15	0.007
GTPBP4	22.44	25.61	25.03	20.38	20.46	17.52	4.91	0.023
UTP11	26.02	27.08	26.98	23.92	22.48	19.52	4.72	0.024
NOP53	24.25	23.02	23.90	18.99	19.42	18.65	4.70	0.000
MICALL2	27.92	28.20	28.36	22.66	25.69	22.62	4.50	0.012
ACACA	23.75	23.39	23.63	20.60	18.21	18.53	4.48	0.004
CTTNBP2NL	24.99	24.86	24.46	19.95	22.05	18.95	4.45	0.009
CCDC141	22.96	23.62	22.77	19.18	18.40	18.44	4.45	0.000
RPL38	22.44	23.56	22.50	19.04	18.32	18.63	4.17	0.001
COL8A1	22.72	22.53	21.72	19.07	18.04	17.72	4.05	0.001
AP3D1	22.43	22.73	23.46	17.67	21.36	17.66	3.97	0.035
TAF2	23.47	24.09	23.72	18.71	21.34	19.32	3.97	0.008
ARID1B	24.16	24.77	24.76	18.37	22.91	21.04	3.79	0.047
PALLD	29.43	29.54	29.95	26.32	26.87	24.98	3.58	0.004
DAZAP1	23.22	22.80	23.01	20.47	18.75	19.12	3.56	0.003
FKBP10	24.73	24.91	25.29	22.22	21.40	20.76	3.52	0.002
ZFP37	25.20	24.83	24.82	21.95	23.34	19.33	3.41	0.045
ZCCHC7	25.55	26.36	26.08	22.15	24.11	21.82	3.31	0.012
LLPH	22.37	23.18	22.64	19.90	19.78	18.71	3.27	0.002
SRSF2	21.34	22.87	22.65	19.12	19.16	18.79	3.26	0.003
LOC108348180	22.85	24.37	21.83	19.19	20.90	19.24	3.24	0.025
ITCH	22.42	22.51	22.00	19.76	18.99	18.57	3.21	0.001
DCP1A	20.95	22.15	21.55	18.38	18.61	18.04	3.20	0.001
LPP	24.76	25.39	26.98	22.92	22.22	22.51	3.16	0.010
RPS15	21.68	22.70	23.08	19.69	19.72	18.59	3.15	0.005
PDLIM1	30.37	30.82	30.60	27.27	27.57	27.52	3.14	0.000
ZSCAN21	23.54	24.83	24.34	20.16	21.28	21.94	3.11	0.008
PEG3	22.56	20.87	22.22	17.93	20.66	17.86	3.07	0.044
LSM14A	22.53	23.86	24.26	20.08	21.68	19.75	3.05	0.018

ATP5IF1	21.76	22.32	23.73	20.37	18.86	19.44	3.05	0.014
ELN	22.17	21.82	20.91	18.28	19.84	17.75	3.01	0.015
SPATA6	22.47	22.40	22.41	19.34	21.08	17.85	3.00	0.033
NOC4L	21.65	22.21	22.22	19.03	19.18	18.92	2.99	0.000
FAM98A	21.83	22.40	21.11	19.20	18.41	18.80	2.97	0.002
PALMD	24.19	24.30	24.65	21.68	22.38	20.24	2.95	0.010
SIPA1L3	23.19	21.90	21.40	18.74	18.75	20.18	2.94	0.015
USP36	22.41	22.52	22.31	18.43	21.21	18.85	2.91	0.028
LOC500684	22.47	23.68	23.34	19.90	22.00	18.92	2.89	0.042
SYNPO	28.98	28.76	28.37	26.28	26.27	25.00	2.86	0.003
LOX	23.03	23.46	22.94	19.26	22.11	19.49	2.85	0.037
FN1	28.89	28.77	30.34	26.40	26.95	26.13	2.84	0.007
NCOA5	21.19	21.80	21.81	18.59	19.99	17.73	2.83	0.015
DDX54	23.44	22.59	22.22	19.34	19.14	21.48	2.77	0.029
SNRPC	23.15	23.10	22.34	19.29	21.68	19.44	2.73	0.029
RBM42	22.36	22.87	22.56	20.23	21.03	18.59	2.65	0.022
ACTN2	28.80	29.21	28.98	26.70	26.79	25.68	2.61	0.002
NKAPD1	23.06	23.30	23.27	19.19	20.76	22.10	2.52	0.041
TP53BP2	21.18	22.15	22.12	17.86	20.39	19.79	2.47	0.040
SIPA1L1	24.04	24.28	23.65	21.15	22.47	21.08	2.43	0.008
COL18A1	28.70	28.86	28.70	27.01	27.19	24.79	2.42	0.035
FHL3	27.27	27.59	27.58	24.82	25.16	25.23	2.41	0.000
CPEB4	23.34	22.57	22.74	21.60	20.43	19.43	2.40	0.023
RPS6	24.50	24.95	24.16	21.92	22.97	21.59	2.38	0.008
PDLIM3	29.72	30.02	29.88	27.60	27.65	27.26	2.37	0.000
ANXA1	25.38	25.42	25.57	23.38	24.19	21.70	2.37	0.032
EIF5A2	20.68	21.67	22.29	18.66	19.15	19.80	2.34	0.015
TNRC6C	21.81	21.35	21.68	19.16	20.26	18.41	2.33	0.014
AMOTL2	21.33	21.88	21.90	19.75	19.79	18.61	2.32	0.006
KNOP1	24.23	24.72	24.25	22.73	22.04	21.50	2.31	0.004
CCDC137	24.18	23.14	23.82	22.20	22.02	20.00	2.31	0.040
GNL2	25.41	25.14	25.55	22.33	23.98	22.88	2.30	0.010
RPL36A	28.75	28.87	28.86	25.78	27.03	26.78	2.30	0.004
LMCD1	20.06	21.08	22.48	19.45	18.82	18.48	2.29	0.039
PHF3	25.57	25.94	26.66	23.58	24.78	23.03	2.26	0.021
PDLIM5	29.38	29.61	29.88	27.71	27.73	26.73	2.24	0.003
RPSA	22.31	22.36	21.82	19.93	20.69	19.23	2.21	0.008
DDX27	24.47	24.74	23.73	22.92	22.41	21.09	2.17	0.026
SHROOM4	21.90	21.52	22.42	18.37	20.69	20.25	2.17	0.046
CHD2	26.02	25.65	26.17	23.56	25.21	22.68	2.13	0.048
UBN1	22.61	22.28	22.79	19.72	21.21	20.50	2.08	0.010
TGFB1I1	25.57	26.21	26.11	24.31	24.56	22.79	2.08	0.024
SAFB2	27.07	27.13	27.52	24.35	26.00	25.21	2.05	0.014

PRRC2A	21.60	20.45	20.85	19.97	18.79	18.00	2.05	0.037
RPS8	25.89	25.87	26.06	23.17	24.48	24.07	2.03	0.007
HTRA1	21.16	20.98	20.61	19.03	19.53	18.16	2.01	0.010
RGD1564606	28.18	28.12	28.30	26.51	26.49	25.62	1.99	0.003
RBPMS	21.34	21.52	21.66	18.77	19.61	20.19	1.99	0.009
RPL26	24.53	23.86	25.01	21.94	23.02	22.48	1.98	0.012
ZFP655	20.88	20.81	21.80	18.70	20.35	18.50	1.98	0.041
ARHGAP21	22.40	21.73	22.69	19.20	21.40	20.30	1.98	0.047
AMOT	20.95	21.16	21.23	18.65	19.80	18.98	1.97	0.005
RRS1	27.15	27.22	27.58	24.95	25.57	25.52	1.97	0.001
ZYX	26.39	26.48	27.14	25.07	24.88	24.20	1.95	0.005
PPIC	22.24	21.81	21.80	18.99	21.15	19.91	1.93	0.040
RPL10	24.90	26.17	25.75	23.12	24.60	23.31	1.93	0.032
PHB2	19.97	21.42	19.73	18.94	18.79	17.65	1.91	0.045
RSL1D1L1	22.06	21.61	21.90	19.33	21.14	19.37	1.91	0.035
ZC3H13	24.91	25.03	25.51	22.71	24.42	22.66	1.89	0.036
MNT	27.35	25.79	26.11	24.19	25.09	24.42	1.85	0.027
NUFIP1	21.19	21.06	21.41	19.42	18.93	19.91	1.80	0.004
PTBP1	25.14	25.07	25.30	23.92	23.44	22.92	1.74	0.004
RBMS2	22.26	21.61	21.94	19.67	20.78	20.15	1.74	0.010
RPL35	27.67	26.66	27.35	26.14	24.95	25.43	1.72	0.020
SSB	25.69	25.81	26.79	24.01	25.24	23.94	1.70	0.036
CNOT1	25.08	25.53	25.18	23.31	24.34	23.13	1.67	0.014
CCDC80	26.07	25.80	26.49	23.75	25.39	24.27	1.65	0.034
DVL3	25.57	25.33	25.57	23.86	24.18	23.49	1.65	0.002
SSX2IP	24.33	24.59	24.46	22.44	23.54	22.62	1.59	0.010
SEC24D	20.46	20.35	20.73	19.65	18.82	18.30	1.59	0.018
ISG20L2	21.58	21.22	20.59	19.49	20.07	19.09	1.58	0.018
LOC100909784	19.81	20.10	19.65	17.66	18.37	18.80	1.57	0.012
RPS23	28.87	28.52	29.05	27.14	27.93	26.67	1.57	0.017
ZFP41	19.03	19.52	19.97	18.74	18.03	17.30	1.48	0.041
LOC681410	23.98	24.18	24.19	21.74	23.39	22.79	1.48	0.038
SMG7	20.42	21.02	21.04	19.29	19.31	19.44	1.48	0.002
DVL2	23.56	23.59	24.22	21.91	22.30	22.78	1.46	0.011
MYOZ2	27.67	27.69	26.95	26.11	26.30	25.65	1.42	0.010
TTN	29.89	29.77	29.77	28.13	28.61	28.52	1.39	0.001
FBL	25.87	26.05	25.89	25.06	24.42	24.17	1.38	0.007
LOC679087	21.71	21.46	21.42	19.55	20.95	19.98	1.37	0.032
RPL18A	24.55	24.77	24.78	24.04	23.32	22.66	1.36	0.029
RPL29	28.64	29.29	28.59	27.50	27.47	27.51	1.35	0.004
TFG	21.04	21.95	21.35	20.52	20.39	19.45	1.33	0.036

MMTAG2	22.21	22.69	22.97	20.79	21.54	21.59	1.32	0.018
RPL3	24.26	24.14	24.38	23.27	23.58	22.19	1.25	0.043
RPL17	25.25	25.24	25.30	23.88	24.55	23.69	1.23	0.009
PPIG	27.95	27.95	27.98	26.34	27.45	26.43	1.22	0.026
NEWGENE_621351	19.91	20.41	21.08	19.06	19.40	19.33	1.20	0.027
TAF3	22.49	23.52	22.75	21.45	22.03	21.79	1.16	0.030
ARGLU1	27.13	26.76	27.63	25.45	26.59	26.04	1.15	0.050
FLNB	29.73	29.90	30.12	28.76	29.06	28.53	1.13	0.004
NOLC1	25.42	25.60	26.07	24.59	25.06	24.09	1.12	0.031
RPS26	25.20	25.10	25.13	23.58	24.47	24.07	1.11	0.013
CHD1	24.46	24.20	24.65	23.08	24.09	22.96	1.06	0.049
SAFB	28.96	28.85	29.26	27.73	28.37	27.87	1.03	0.011
RPS27A	28.67	28.71	28.57	27.43	27.79	27.64	1.03	0.001
PICALM	26.81	26.66	26.85	25.37	26.25	25.61	1.03	0.018
HNRNPU	30.47	30.20	30.24	29.51	29.55	28.83	1.01	0.015

Supplemental Table 2. sgRNAs

Species	Target	Top strand (5'-3')	Bottom strand (5'-3')
Mus musculus	None	ACCGGCGAGGTATTCGGCTCCGCG	AAACCGCGGAGCCGAATACCTCGC
Mus musculus	Rpsa	ACCGGGTTTATGATGTAGATACCT	AAACAGGTATCTACATCATAAACC
Mus musculus	Rpsa	ACCGCAAGTACCTTGTTGTTGCAT	AAACATGCAACAACAAGGTACTTG
Mus musculus	Rpsa	CTTACAGGGAGCTCACTCAG	CTGAGTGAGCTCCCTGTAAG

Supplemental Table 3. Antibodies

Name	Manufacturer	Catalog #
Anti-Cardiac Troponin I antibody	Abcam	ab47003
Anti-alpha-Actinin antibody	Sigma-Aldrich	A7811
Anti-BioID2 antibody [SS 3A5-E2]	Abcam	ab232733
Anti-67kDa Laminin Receptor antibody [EPR8469]	Abcam	ab133645
Anti HA-tag antibody	Abcam	ab9110
Anti HA-tag antibody	Cell signaling Technology	3724
Anti MYBPC3 antibody	Abcam	Ab262964
Anti RPS6 antibody	Cell Signaling Technology	C-2217
Anti RPL10A antibody	Abcam	ab174318
Anti-alpha TUBULIN antibody	Cell Signaling Technology	3873S
Anti-MYH antibody	DSHB	A4.1025
Donkey Anti-Rabbit IgG antibody IRDye® 680RD	Li-Core Bioscience	926-68073
Goat Anti-Mouse IgG Antibody, IRDye® 800CW	Li-Cor Bioscience	926-32210
Lectin from Triticum vulgaris (wheat), FITC conjugate	Sigma Aldrich	L4895
Click-iT TUNEL Alexa Fluor 488 Imaging Assay	Thermo-Fisher Scientific	C10245
Anti VDAC1 antibody	Abcam	Ab154856
Anti GAPDH antibody	Sigma Aldrich	G8795
Anti SDHA antibody	Cell Signaling Technology	11998
