# Supplementary material for: Localized translation and sarcomere maintenance requires ribosomal protein SA in mice
Source: J Clin Invest. 2024 May 14;134(13):e174527. doi: 10.1172/JCI174527 (PMC11213506; doi:10.1172/JCI174527)

Figure 3B (RPSA)

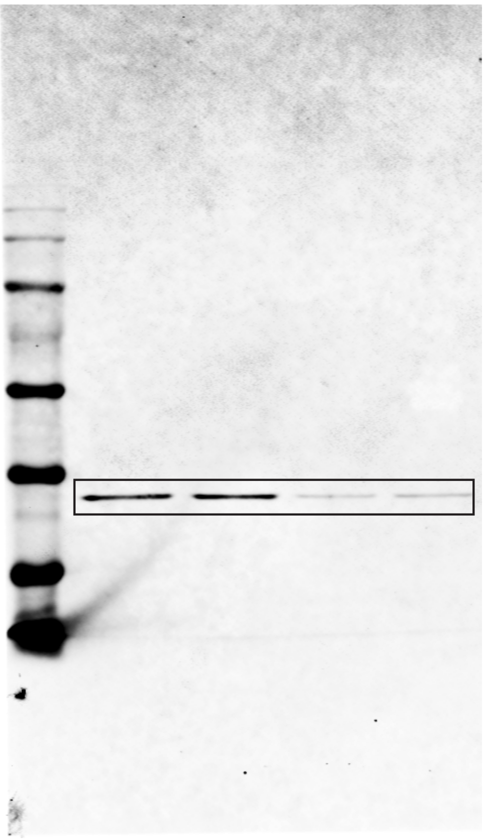

Supplemental Figure 2B

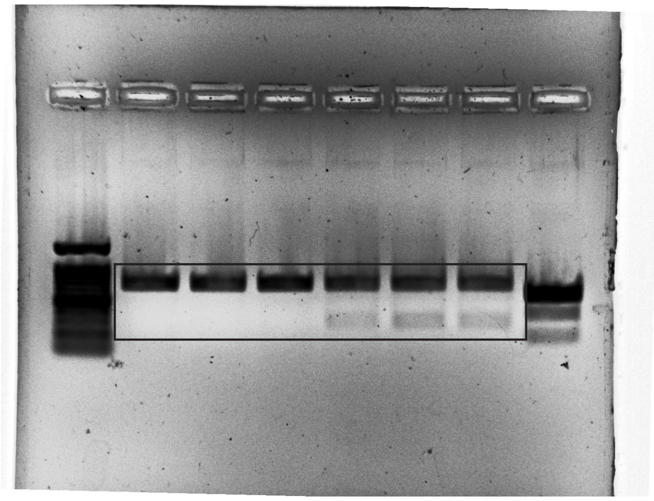

Supplemental Figure 2C

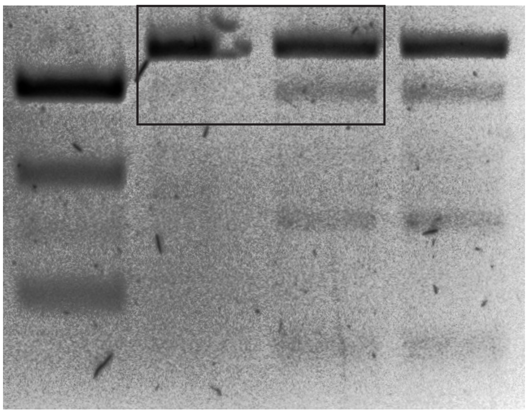

Supplemental Figure 4A

Replicate 1

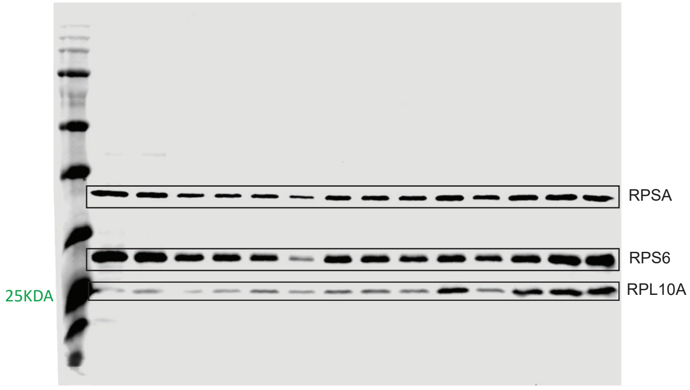

Replicate 2

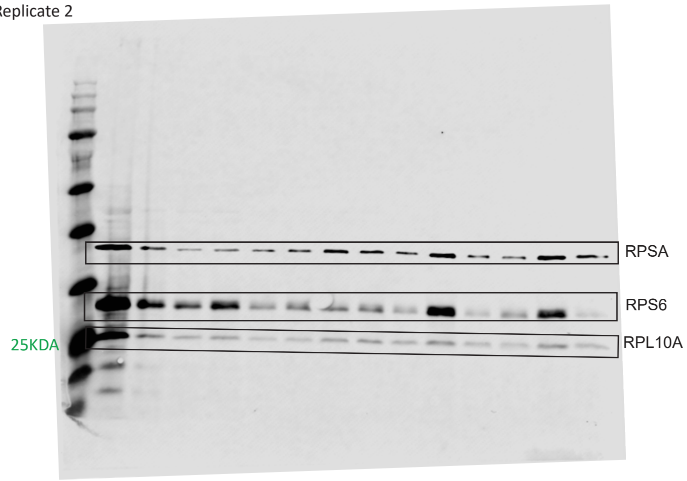

Figure 6K
anti-α Tubulin-Ab.

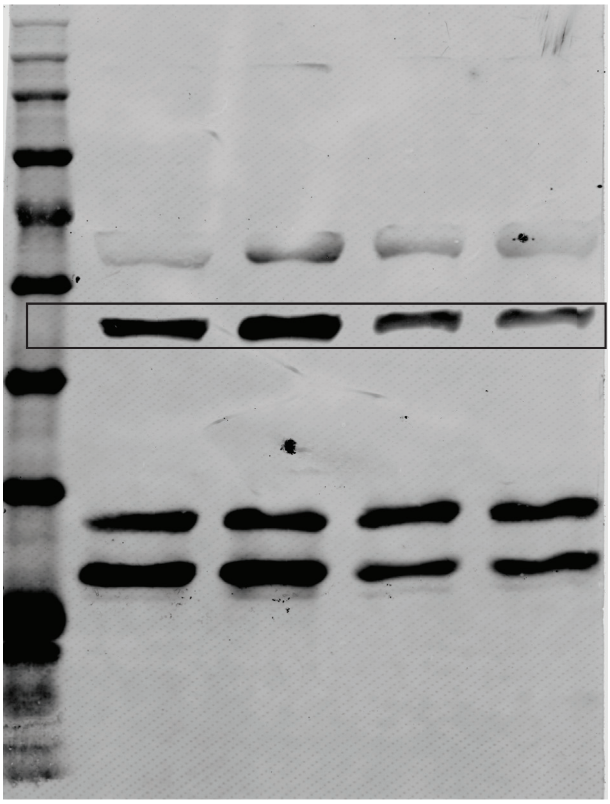

Figure 6K
anti-VDAC1-Ab.

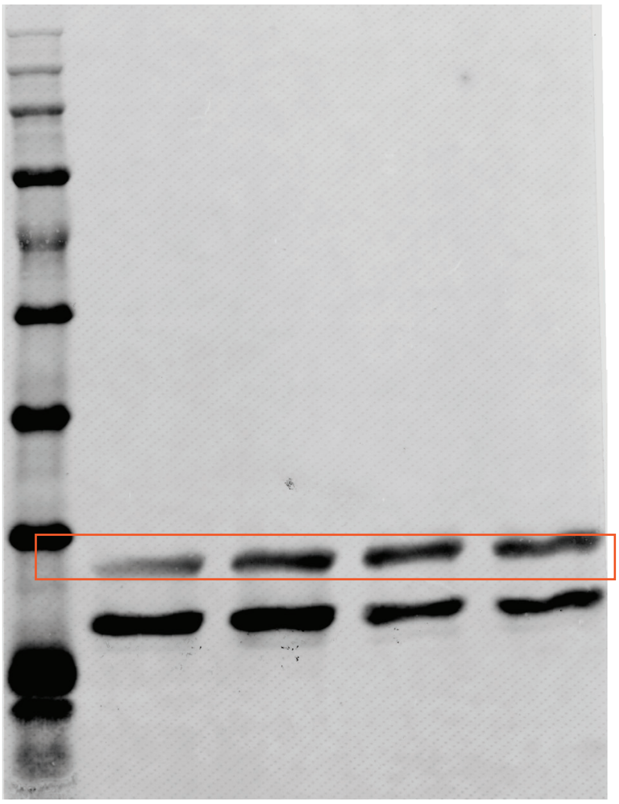

Figure 6K
anti-GAPDH-Ab.

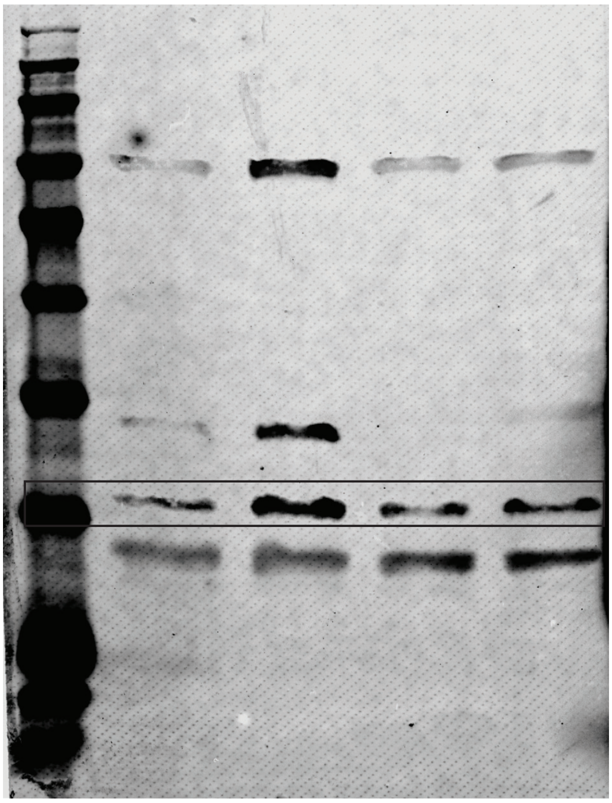

Figure 6K
anti-SDHA-Ab.

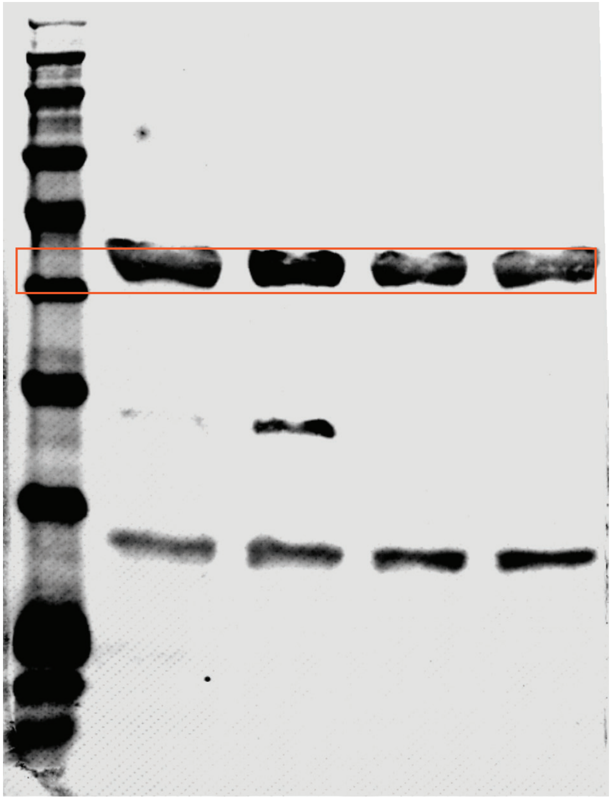

Figure 6K
anti-RPSA-Ab.

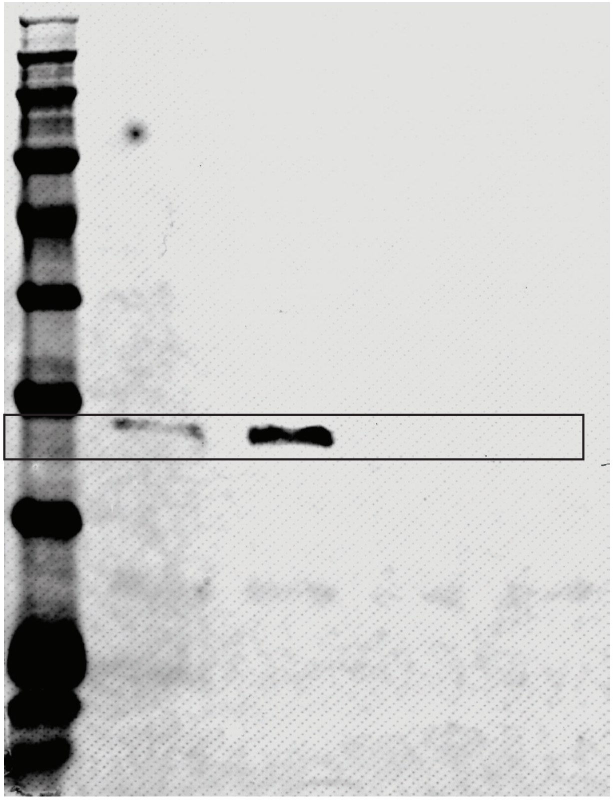

Figure 6K
anti-ACTN-Ab.

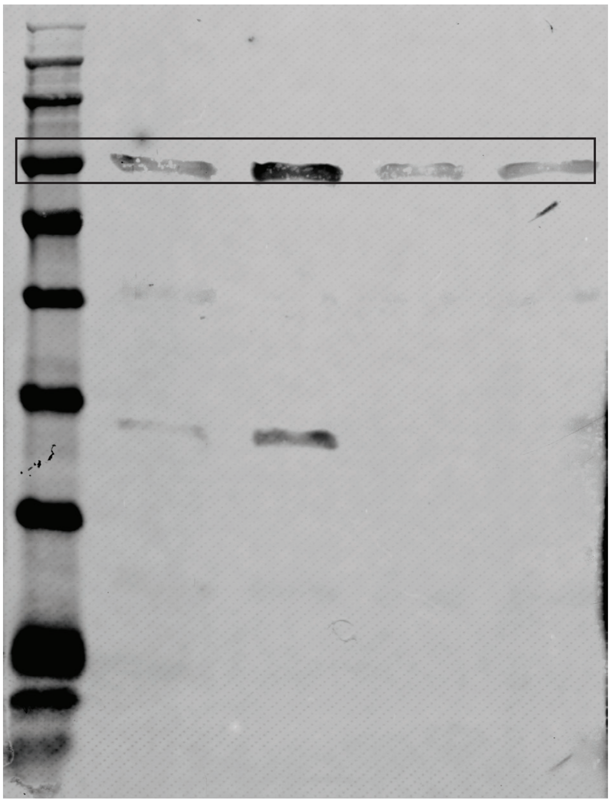

Figure 6K
anti-MYBPC3-Ab.

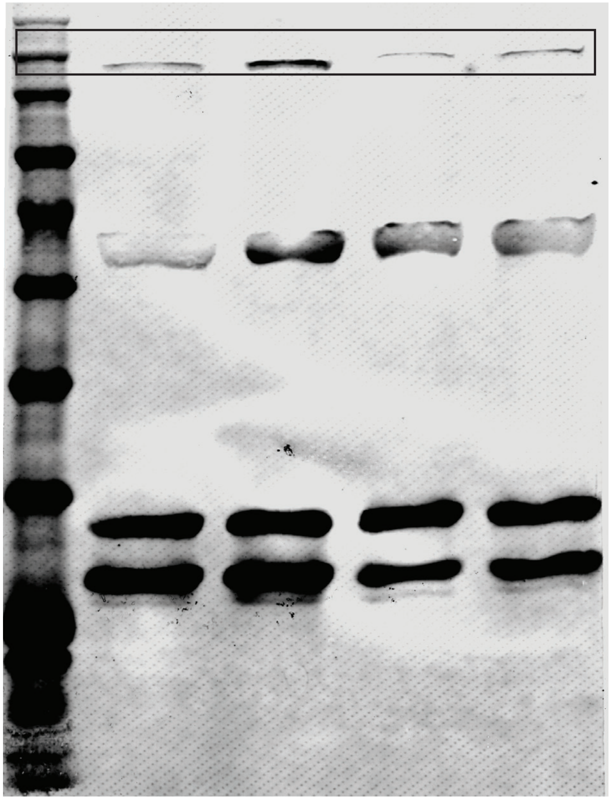

Figure 6K
anti-TNNI-Ab.

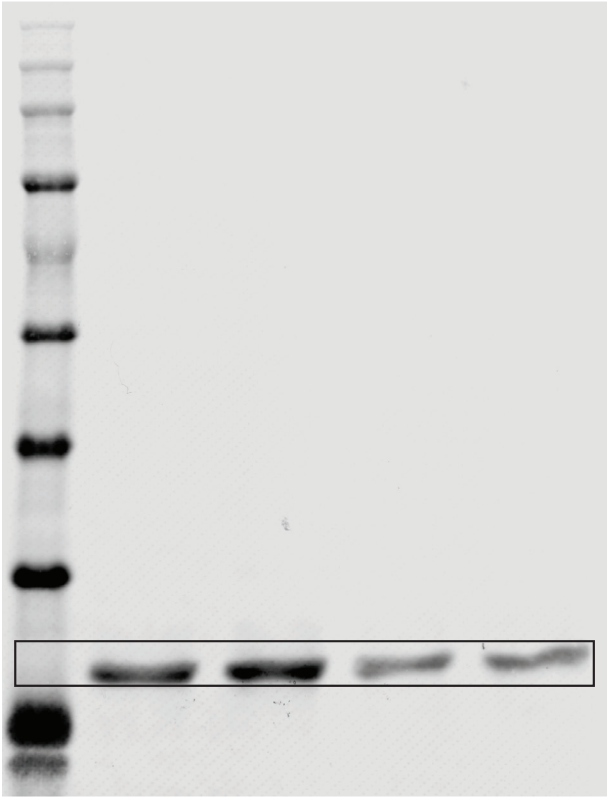

Supplement: Unedited blot and gel images [file jci-134-174527-s033.pdf]
